# DFASGCNS: A prognostic model for ovarian cancer prediction based on dual fusion channels and stacked graph convolution

**DOI:** 10.1371/journal.pone.0315924

**Published:** 2024-12-16

**Authors:** Huiqing Wang, Xiao Han, Shuaijun Niu, Hao Cheng, Jianxue Ren, Yimeng Duan

**Affiliations:** College of Computer Science and Technology (College of Data Science), Taiyuan University of Technology, Taiyuan, China; State University of New York at Oswego, UNITED STATES OF AMERICA

## Abstract

Ovarian cancer is a malignant tumor with different clinicopathological and molecular characteristics. Due to its nonspecific early symptoms, the majority of patients are diagnosed with local or extensive metastasis, severely affecting treatment and prognosis. The occurrence of ovarian cancer is influenced by multiple complex mechanisms including genomics, transcriptomics, and proteomics. Integrating multiple types of omics data aids in predicting the survival rate of ovarian cancer patients. However, existing methods only fuse multi-omics data at the feature level, neglecting the shared and complementary neighborhood information among samples of multi-omics data, and failing to consider the potential interactions between different omics data at the molecular level. In this paper, we propose a prognostic model for ovarian cancer prediction named Dual Fusion Channels and Stacked Graph Convolutional Neural Network (DFASGCNS). The DFASGCNS utilizes dual fusion channels to learn feature representations of different omics data and the associations between samples. Stacked graph convolutional network is used to comprehensively learn the deep and intricate correlation networks present in multi-omics data, enhancing the model’s ability to represent multi-omics data. An attention mechanism is introduced to allocate different weights to important features of different omics data, optimizing the feature representation of multi-omics data. Experimental results demonstrate that compared to existing methods, the DFASGCNS model exhibits significant advantages in ovarian cancer prognosis prediction and survival analysis. Kaplan-Meier curve analysis results indicate significant differences in the survival subgroups predicted by the DFASGCNS model, contributing to a deeper understanding of the pathogenesis of ovarian cancer and providing more reliable auxiliary diagnostic information for the prognosis assessment of ovarian cancer patients.

## Introduction

Ovarian cancer is a tumor with a range of distinct clinicopathological and molecular features [1]. Due to its inconspicuous early symptoms, it is often diagnosed at an advanced stage, leading to high recurrence rates and low survival rates for patients [2,3]. The occurrence of ovarian cancer is influenced by complex mechanisms at multiple levels including genomics, transcriptomics, and proteomics [4–6], and different types of omics analysis aid in predicting the survival rate of ovarian cancer patients [4,6,7]. Ovarian cancer is a heterogeneous disease characterized by molecular and omics diversity. While single omics data focus on specific molecular aspects of ovarian cancer, integrating different omics data can provide complementary information from different molecular perspectives. Comprehensive analysis of multi-omics data is crucial for understanding the pathogenesis of ovarian cancer and predicting patient prognosis. Boehm et al. [8] integrated histopathological, radiological, and clinical genomic data of high-grade serous ovarian cancer to predict patient prognosis using a risk stratification model. Zhang et al. [9] utilized multi-omics data including gene expression, somatic DNA alterations, miRNA expression, and DNA methylation in serous ovarian cancer (SOC) to determine patient prognosis and treatment efficacy, offering new insights for improved treatment strategies. Therefore, ovarian cancer prognosis prediction based on multi-omics data not only improves prediction accuracy but also deepens researchers’ understanding of the biological characteristics and molecular mechanisms of ovarian cancer. This provides valuable insights for clinical practice and personalized treatment strategies for patients.

Due to different omics data providing varying perspectives on ovarian cancer, the integration of multi-omics data can comprehensively reveal the molecular characteristics of ovarian cancer diversity from the aspects of genomics, transcriptomics and epigenomics [10], contributing to a more comprehensive understanding of the potential biological processes underlying ovarian cancer development [11,12]. Existing researchers mainly fuse multi-omics data through two methods: feature-based fusion and graph-based fusion. Feature-based fusion methods integrate the feature representations of different omics data into a unified feature space to reveal the inherent correlations among multi-omics features. For example, Cheerla et al. [13] utilized unsupervised encoders to compress clinical data, mRNA expression, microRNA expression, and histopathology whole slide images (WSIs) into 512-dimensional feature vectors. They aggregated the four feature vectors into a common feature space using similarity loss for predicting the survival rate of pancreatic cancer patients. The GPDBN method [14] and HBSurv method [15] employed a bilinear feature encoding module to fuse the features of multi-omics data and clinical data into a feature space, effectively utilizing relationships between and within modalities to enhance the predictive performance of the model. Graph-based multi-omics fusion methods represent different types of omics data as graph structures, where nodes represent cancer samples and edges represent relationships between samples. These methods fuse the graph structures of different omics data to achieve comprehensive analysis across omics data types, fully considering the correlations between samples of different omics data. The GCGCN method [16] fused the sample-sample similarity matrices of multi-omics data (including gene expression, copy number alteration, DNA methylation, and exon expression) and clinical data into a sample network graph structure using a similar network fusion algorithm (SNF). It obtained shared and complementary information from different data sources and achieved survival prediction for Breast cancer (BRCA) and Lung squamous cell cancer (LUSC). Wang et al. [17] used cosine similarity to construct a weighted sample similarity network for cancer multi-omics data (mRNA, DNA methylation, and miRNA), and classified cancer subtypes by learning the correlation between omics data and samples through graph convolutional network. Although these methods outperform single-omics approaches, feature-based fusion methods only focus on low-dimensional feature representations of multi-omics data, while graph-based methods prioritize the associations between cancer samples. Therefore, this paper proposes a dual fusion channel strategy that combines feature fusion and graph fusion. The strategy not only fully learns the feature representations of different omics data at the feature level but also considers the inherent correlations between ovarian cancer samples, obtaining more reliable information for prognosis prediction of ovarian cancer patients.

In recent years, researchers have used deep learning methods for cancer subtype classification and patient prognosis prediction based on multi-omics data by learning local features of each omics data type and directly concatenating the local features of various omics data for cancer classification or prediction. Liu et al. [18] employed convolutional autoencoders (CAE) to extract low-dimensional feature representations of RNA and CNV separately, concatenated them, and used a univariate Cox proportional hazards (Cox-PH) model to select features associated with cancer survival for patient prognosis prediction. DeepMO [19] learned local features of mRNA, DNA methylation, and CNV through corresponding encoding subnetworks, concatenated these features as inputs to a classification subnetwork, and achieved breast cancer subtype classification. ConcatAE [20] used autoencoders (AE) to learn latent variable features of different types of omics data, including Gene Expression, DNA Methylation, miRNA Expression, and CNV, and concatenated them for survival prediction of breast cancer patients. However, these methods learn local features of multi-omics data and fuse the features of multi-omics data directly by concatenation, neglecting the mutual influence of different omics data at the molecular level and failing to consider the direct or indirect relationships among samples, thus failing to comprehensively learn the interactions and potential structures among multi-omics data, which reduces the interpretability and generalization of the model. Recently, graph convolutional network (GCN) have shown promising performance in various fields such as cancer patient survival prediction and cancer subtype classification by simultaneously leveraging both omics features and the similarity networks describing correlations among samples [21]. Ling et al. [22] proposed the survival model AGGSurv based on GCN, constructing different sparse graphs using random subsets of multi-omics high-dimensional features, which aids in survival analysis of cancer patients. However, traditional GCN can only capture the direct neighbor information of nodes in a single convolution operation, neglecting the potential network structure among the nodes, which limits the extraction of global information among different omics data. Therefore, this paper proposes Stacked Graph Convolutional Network (SGCN), which builds upon a single-layer GCN by stacking multiple layers of graph convolutions. This progressively expands the receptive field, allowing for the indirect capture of relationships between more distant nodes, thereby extracting global features among multi-omics data. By stacking multiple graph convolutional layers, this paper integrates features of all multi-omics data and relationships among samples, effectively learning the local and global complementary information of ovarian cancer multi-omics data, thereby improving the accuracy of the model for prognosis prediction.

Attention mechanisms regularize feature maps and word embeddings, allowing deep learning models to focus more on specific regions relevant to the model’s objectives [23–25]. Research has shown that there exist important features highly correlated with prognosis and patient survival in cancer multi-omics data [26–28]. By utilizing attention mechanisms to focus on key features and optimize the learning process of these critical features, the accuracy and interpretability of models can be effectively improved. Choi et al. [29] developed the breast cancer subtype classification model moBRCA-net, which uses attention modules to learn the importance of features from different omics data, enhancing classification performance. Sanghyuk et al. [30] proposed the Multi-Prognosis Estimation Network (Multi-PEN), which employs gene attention layers for both mRNA expression and miRNA expression, identifying genes associated with the prognosis of low-grade glioma (LGG). Prognostic genes play crucial roles in cancer regulation, and the selection and attention to prognostic genes significantly impact the survival status of patients. Therefore, this paper introduces attention mechanisms to select important features closely related to ovarian cancer prognosis from multi-omics data, and assigns corresponding attention weights to the features according to their importance, so as to optimize the feature representations in order to improve the model’s prognosis prediction performance.

In this study, we propose the deep learning model DFASGCNS, which uses a dual fusion channel and stacked graph convolutional network for predicting the prognosis of ovarian cancer patients. The DFASGCNS model proposed a dual fusion channel strategy to select and effectively learn the feature representations of various omics data, merging them at the feature level while constructing graph structures corresponding to different omics data. This approach enables an in-depth analysis of the associations and potential network structures between samples of different omics data, thereby revealing the complex interactions among them. Stacked graph convolutional network (SGCN) is proposed, where a single GCN layer learns the feature representations and direct neighborhood information of multi-omics data, while multiple stacked GCN layers explore the deeper connections and complex relational networks among multi-omics samples. This comprehensive capture of feature representations and sample associations enhances the overall understanding of the data. Attention mechanism is introduced to select important genes related to ovarian cancer prognosis, assigning corresponding attention weights based on feature importance, focusing the model more on features highly relevant to prognosis prediction. The experimental results show that compared with the existing methods, the model DFASGCNS in this paper fully considers the feature representation of different omics data and the association between samples, comprehensively acquires the complementary information and deep association of multi-omics data, improves the performance of ovarian cancer prognosis prediction, and contributes to the understanding of the pathogenic mechanism of ovarian cancer and the identification of personalized treatment plans for patients.

## Materials and methods

### Data collection

This paper downloaded multi-omics data and clinical data of ovarian cancer from the TCGA database (https://portal.gdc.cancer.gov/). The omics data includes mRNA expression, DNA methylation, miRNA expression and copy number variation (CNV)), the corresponding number of features are 46610, 24923, 1874 and 24740 respectively. The details are shown in Table 1. The clinical data describes the clinical information of 587 ovarian cancer patients, including age, race, FIGO stage, survival time, survival status and other characteristics.

**Table 1:** The summary of ovarian cancer data.

Omics type	Number of samples	Number of features	Summary
mRNA	367	46610	HTSeq-FPKM
DNA methylation	363	24923	Illumina Human Methylation 27k
miRNA	499	1874	BCGSC Illumina HiSeq
CNV	606	24740	Affymetrix SNP Array 6.0

### Data preprocessing

This paper preprocesses the downloaded data using a 4-step methods. The first step is to intersect the samples of the mRNA expression, DNA methylation, miRNA expression and CNV data sets to obtain 325 common samples in the cross dataset. Secondly, to filter the features with more than 20% missing values and combine the expression values genes with ’0’ were converted to ’NA’, and the R package ‘ImputeMissings’ [31] was used to fill missing values based on the median. Thirdly, due to the large number of omics data features, the variance threshold method was applied to select the features with variance, calculated over all patients, higher than the given threshold [32,33], the variance thresholds of mRNA, DNA methylation and CNV were initially determined to be 7, 0.02 and 0.1 respectively. Finally, we use z-score techniques to normalize data. After preprocessing, the number of features for mRNA, DNA methylation, miRNA and CNV are 8492, 6125, 454, and 2274 respectively, and the number of samples is all 325, as shown in Table 2.

**Table 2:** Summary information of the multi-omics data of ovarian cancer after preprocessing.

Omics type	Number of samples	Number of features	Summary
mRNA	325	8492	HTSeq-FPKM
DNA methylation	325	6125	Illumina Human Methylation 27k
miRNA	325	454	BCGSC Illumina HiSeq
CNV	325	2274	Affymetrix SNP Array 6.0

### Model architecture

**Fig 1:**
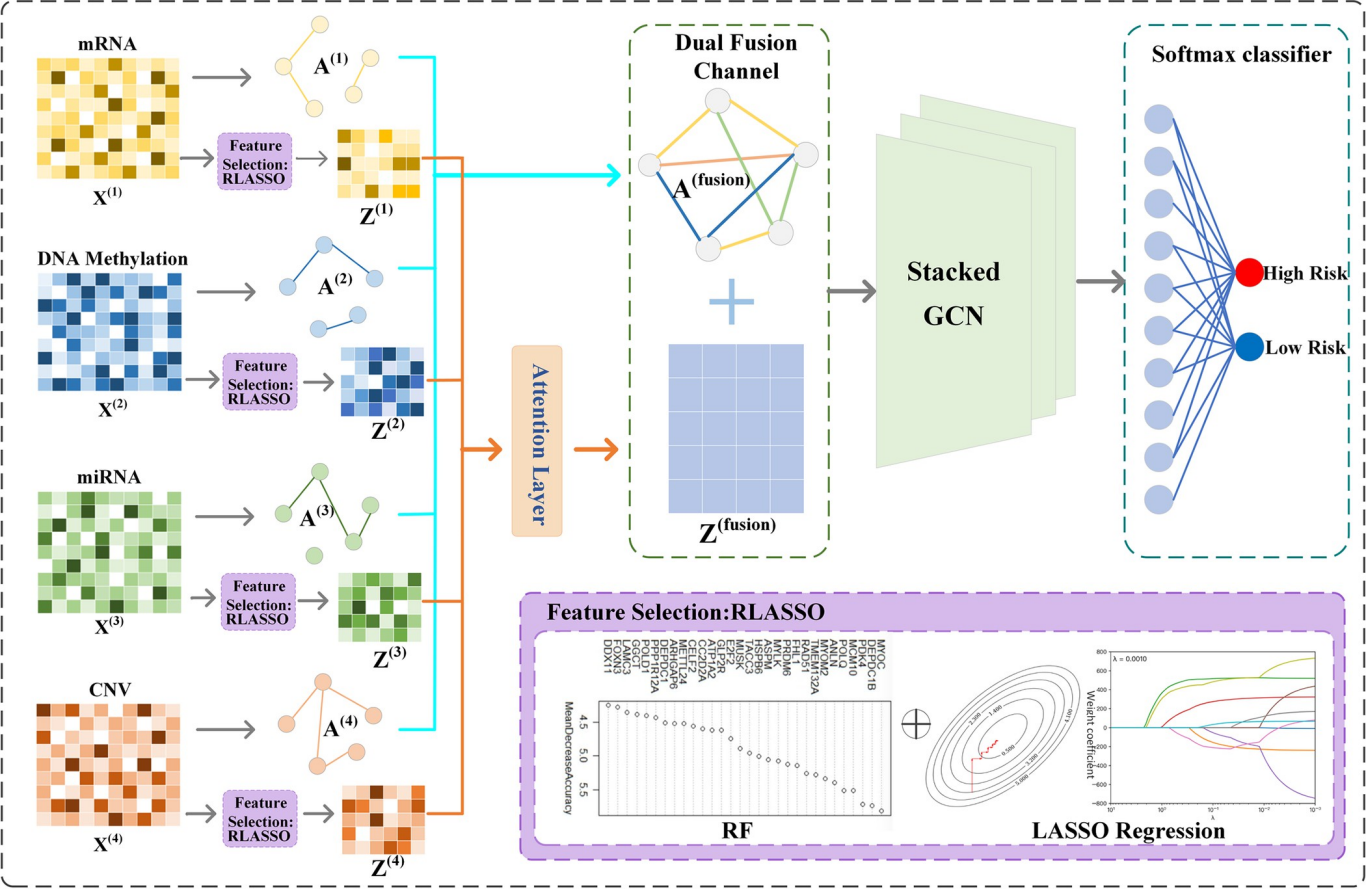
The architecture of DFASGCNS.

To leverage the complementary information of different omics data and fully consider the shared and neighborhood information among different omics samples, this paper proposes a novel ovarian cancer prognosis prediction model, DFASGCNS. This model is based on stacked graph convolution network (SGCN) and employs a dual fusion channel strategy, aimed at simultaneously learning the omics feature data and the intrinsic correlations between samples. Specifically, DFASGCNS preprocesses ovarian cancer’s mRNA expression, DNA methylation, miRNA expression, and CNV as inputs, selects genes closely related to ovarian cancer prognosis through a feature selection algorithm, and employs an attention mechanism to allocate different weights to important features across different omics data, optimizing the representation of key features in various omics data. For each type of omics data, a relational graph between samples is constructed, and these graphs are fused into a unified graph structure. The fused graph, together with the fusion feature representation weighted by the attention mechanism, is input into the SGCN to comprehensively learn the feature information of the ovarian cancer multi-omics data and the sample network structure. Finally, the prognosis of ovarian cancer patients is predicted using a softmax classifier. The architecture of the DFASGCNS model is depicted in Fig 1.

#### Feature selection method: RLASSO

Ovarian cancer multi-omics data are characterized by low sample size and high dimensionality. Feature selection methods can effectively capture important features from the high-dimensional data, thereby enhancing the predictive performance of the model [34]. As shown in Table 2, the number of features after preprocessing for mRNA, DNA methylation, miRNA, and CNV are 8492, 6125, 454, and 2274, respectively. To identify features highly correlated with ovarian cancer prognosis, this study introduces the use of a random forest and the RLASSO feature selection method. Important features identified by the random forest serve as supplements to the features lost during LASSO regression. The final counts of features for each type of omics data are shown in Table 3.

**Table 3:** Summary information of the multi-omics data of ovarian cancer after RLASSO.

Omics type	Number of samples	Number of features
mRNA	325	143
DNA methylation	325	142
miRNA	325	128
CNV	325	136

In RLASSO, initially, LASSO regression employs L1 regularization by adding a penalty term to the least squares error component in the objective function. During the optimization process, this causes the coefficients of some features to shrink towards zero, and ultimately, reduces the coefficients of certain features entirely to zero, thereby facilitating feature selection. The formula for feature selection using LASSO regression is as follows:

$$\begin{array}{c} Min\sum_{j=1}^{N}{\left(y_{j}^{i}-\sum_{k=1}^{d^{i}}x_{jk}\omega_{k}\right)}^{2}+\lambda \sum_{k=1}^{d^{i}}{\left|\left|\omega_{k}\right|\right|}_{1}\\ subject\;to:\sum_{k=1}^{d^{i}}{\left||\omega_{k}|\right|}_{1}<c \end{array}$$ (1)

Where, *i* represents the *i*-th omics data type, *N* denotes the number of samples, *d*^*i*^ indicates the total number of features for the *i*-th omics data, *y*_*j*_ represents the label of the *j*-th sample, and *λ* is the regularization parameter.

Importance ranking of all features using random forest to construct a decision tree, and the first *K* features with high importance are selected based on the criteria of feature importance. In the feature set of omics data $F=\{f_{1},f_{2},f_{3},\ldots ,f_{\left(d^{i}\right)}$}, the feature importance collection $I=\{I_{1},I_{2},I_{3},\ldots ,I_{\left(d^{i}\right)}$} is outputted based on feature importance. Here, *d*^*i*^ represents the total number of features for the *i*-th omics data, and the formula for *I*_*x*_ is as follows:

$$I_{x}=\frac{1}{N}\sum_{k=1}^{N}\left(R_{n}^{oob}-R_{nj}^{oob}\right)$$ (2)

Where, $R_{p}^{oob}$ and $R_{pj}^{oob}$ represent data outside of the bag before and after the decision tree disturbance (i.e. samples not sampled during decision tree resampling), and the number of correctly classified samples is tallied. The top *K* features are selected in descending order of importance for feature selection. In this study, the optimal predictive performance is achieved when *K* = 100. The important features selected by random forest and the features retained by LASSO regression are combined as the total features for specific omics data.

#### Construction and fusion of relationship diagrams between samples

While feature fusion can capture the complementary information at the feature level from different omics data for each patient, it overlooks the shared and complementary neighborhood information among samples from different omics data. Addressing this issue, the DFASGCNS model integrates multiple graphs constructed from ovarian cancer single omics data and generates a fused graph, accurately revealing the intrinsic correlations among samples. Given the data representation $\{x_{i}{\}}_{i=1}^{n}$ for *n* samples, where *ρ*(*x*_*i*_,*x*_*j*_) represents the Euclidean distance between samples *x*_*i*_ and *x*_*j*_, we construct the adjacency matrix *A* of the *k*-nearest neighbor graph using the exponential similarity kernel. Where, *A*∈*R*^*n*×*n*^, and the calculation process is as follows:

$$A(i,j)=\{\begin{array}{cc} exp\left(\frac{-\rho^{2}(x_{i},x_{j})}{\mu \delta^{2}}\right), & j\in N_{i}\\ 0, & \mathrm{otherwise} \end{array}$$ (3)

Where *N*_*i*_ represents the *k* nearest neighbors of sample *i*,*δ*^2^ is used to address scale issues and is experimentally set to the median of the squared distances between all pairs of samples. *μ* is the hyperparameter, which is set to 0.5.

According to the construction method of the *k*-nearest neighbor graph described above, four single omics graphs were built using mRNA expression data *X*^(1)^, DNA methylation data *X*^(2)^, miRNA expression data *X*^(3)^, and CNV data *X*^(4)^, respectively denoted as *A*^(1)^、*A*^(2)^、*A*^(3)^ and *A*^(4)^. These graphs were fused by taking the average of the four adjacency matrices to obtain the final fusion graph of adjacency matrix *A*.

#### Introducing attention mechanism for feature fusion

In ovarian cancer, different omics data have varying degrees of importance in prognosis prediction [37], such as mRNA expression, DNA methylation, miRNA expression, and CNV. The self-attention mechanism, through the training process, can autonomously learn the feature weights of each omics data type and adaptively evaluate the importance of each feature, enabling feature weighting. Compared to traditional static feature selection methods, the self-attention mechanism better captures the interdependencies and hidden relationships between multi-omics data. Through feature weighting and fusion, it enhances the model’s focus on key features, ultimately improving the performance and accuracy of prognosis prediction. Therefore, this study introduces the self-attention mechanism to explore the contribution of different omics data to ovarian cancer prognosis prediction. By assigning different weights to each type of omics data, the model can better represent the important features during feature fusion, thus improving the representation of crucial multi-omics data. After preprocessing, mRNA expression, DNA methylation, miRNA expression, and CNV data through RLASSO feature selection, resulting in low-dimensional representations denoted as $Z^{\left(k\right)}\in R^{n\times d_{k}}$, where *k* = 1,2,3,4 represents the *k*-th omics data type, *n* denotes the number of samples. *d*_*k*_ denotes the number of features for the *k*-th omics data. Each omics data type is treated as a feature matrix where each row corresponds to a sample and each column corresponds to a feature. To explore the contribution of different omics data to prognosis prediction, this paper introduces a self-attention mechanism that utilizes adaptive attention weights to assign importance to the features of various omics data, thereby enhancing the model’s prognostic performance. Specifically, an attention weight vector $\alpha^{\left(k\right)}\in R^{d_{k}}$ is introduced for important features of each omics data, representing the model’s attention to each feature and its importance in the fusion process. The calculation process of attention weights *α*^(*k*)^ is as follows:

$$\alpha^{\left(k\right)}=\mathrm{softmax}(W^{\left(k\right)}h^{\left(k\right)}+b^{\left(k\right)})$$ (4)

Where, $W^{\left(k\right)}\in R^{d_{k}\times d}$ represents the learnable weight matrix, *b*^(*k*)^∈*R*^*d*^ represents the bias vector, $h^{\left(k\right)}\in R^{d_{k}}$ represents the feature representation of the *k*-th omics data, and *d* denotes the number of hidden units in the attention mechanism. The attention weight vector *α*^(*k*)^ is multiplied with the feature representation *Z*^(*k*)^ to obtain the attention-weighted omics feature representation. Consequently, the fused feature representation *Z*^(*fusion*)^ is obtained. The calculation process is as follows:

$$Z^{\left(fusion\right)}=\sum_{k=1}^{4}\;\alpha^{\left(k\right)}Z^{\left(k\right)}$$ (5)

Where *Z*^(*k*)^ represents the feature representation of the *k*-th omics data, and *α*^(*k*)^ is the attention weight vector for the corresponding omics data.

#### Stacked graph convolutional network (SGCN)

To extract deeper and complex network of relationships for survival prediction from ovarian cancer multi-omics data, this study proposes a dual fusion channel strategy to merge feature representations between omics data and relationships among samples to obtain richer features. Stacked Graph Convolutional Network (SGCN) is proposed to learn the fused graph *A* and the global feature representation *Z*^(*fusion*)^ after the fusion of multi-omics data. In SGCN, graph convolutional network is a deep learning model for processing graph-structured data. When calculating the convolution matrix, the graph structure needs to be modeled first. Specifically, the procedure of calculating the convolution matrix $\hat{A}$ in SGCN is shown below:

$$\hat{A}={\tilde{D}}^{-\frac{1}{2}}\tilde{A}\;{\tilde{D}}^{-\frac{1}{2}}$$ (6)

Where $\tilde{A}=A+I_{n},I_{n}$ is the *n*-order identity matrix. $\tilde{D}=diag\;({\tilde{d}}_{1},{\tilde{d}}_{2},\ldots ,{\tilde{d}}_{n})$ represents the degree matrix derived from $\tilde{A}$, where ${\tilde{d}}_{i}=\sum_{j}\;{\tilde{A}}_{ij}$. For the node representation matrix *Z*^(*fusion*)^ = [*z*_1_,*z*_2_,…,*z*_*n*_] with *n* samples and the convolution matrix $\hat{A}$, in the stacked graph convolution network, the calculation of each graph convolutional layer’s output is as follows:

$$Z^{\left(l+1\right)}=f_{2}(f_{1}(\hat{A}\;Z^{\left(fusion\right)}W_{1}^{\left(l\right)})W_{2}^{\left(l\right)})$$ (7)

Where $W_{1}^{\left(l\right)}$ and $W_{2}^{\left(l\right)}$ are the weight matrices of the *l*-th graph convolutional layer, *f*_1_ and *f*_2_ are tanh and sigmoid activation functions, respectively. Repeated the calculation process of the graph convolutional layers, experimental validation shows that the optimal predictive performance of the model is achieved when the number of convolutional layers is set to 5.

Through SGCN, the fused omics data features and the relationship graph among samples are learned, with the output *Z*^*h*^ of the last layer of the graph convolutional network serving as the final high-level feature input to the softmax classifier. For a given layer *i*, this study employs Relu as the activation function between the input data *Z*^*h*^ and the output layer *y*, with the calculation process as follows:

$$y=f_{i}(Z^{h})=relu(W_{i}(Z^{h})+b_{i})$$ (8)

Where *Z*^*h*^ and *y* are two vectors of length *l* and *q*, respectively. *W*_*i*_ represents the weight matrix of size *l*×*q*, and *b*_*i*_ is a bias vector of length *q*. The cross-entropy function is utilized as the loss function, with the calculation process as follows:

$$Cross-entropy=-\frac{1}{n}\sum_{i=1}^{n}\;[y_{i}\cdot log(p_{i})+(1-y_{i})\cdot log(1-p_{i})]$$ (9)

Where *y*_*i*_ represents the label of sample *i*, with high risk being 1 and low risk being 0, and *p*_*i*_ represents the predicted probability of sample *i*.

### Model training

In this study, DFASGCNS was implemented based on Torch 1.10.0 and Python 3.6.11. During the training process, the learning rate, number of epochs, and batch size were set to 0.001, 1000, and 32, respectively. The Adam algorithm was utilized to optimize the objective function. To prevent overfitting, dropout and weight decay (L2 regularization) were implemented to ensure the effectiveness of the model, with dropout rate and weight decay rate set to 0.2 and 0.001, respectively.

### Evaluation metrics

In this paper, the performance of DFASGCNS in predicting ovarian cancer prognosis was evaluated using the following evaluation metrics: accuracy (ACC), F1-score, and the area under the receiver operating characteristic curve (AUC). Accuracy (ACC) is defined as:

$$ACC=\frac{TP+TN}{TP+TN+FP+FN}$$ (10)

where TP, TN, FP, and FN represent true positives, true negatives, false positives and false negatives, respectively. The F1-score is a weighted average of precision and recall, and it is defined as:

$$F1-score=\frac{2\times precision\times recall}{precision+recall}$$ (11)

where precision represents the percentage of accurately predicted positive samples out of all positive samples, and recall represents the rate of accurately predicted positive samples out of all accurate positive samples. They are defined as follows:

$$precision=\frac{TP}{TP+FP}$$ (12)

$$recall=\frac{TP}{TP+FN}$$ (13)

The AUC is the area under the ROC curve. The larger the area, the better the prediction effect of the model.

## Results

### Selection of k value in k-neighbor graph

**Fig 2:**
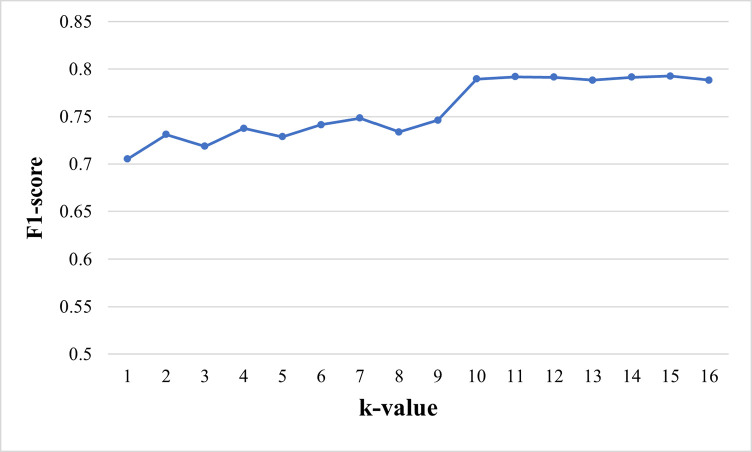
The results of ovarian cancer prognosis prediction with different values of k.

The hyperparameter k in the model DFASGCNS represents the size of the neighborhood in the k-nearest graph. Its selection is determined through cross-validation on the training data. To assess its robustness, we trained DFASGCNS using different k values within a relatively large range. We set the range of k values from 2 to 16, as depicted in Fig 2. Despite variations in the F1-score with changing k values, DFASGCNS demonstrated strong robustness, particularly within the range of k values from 10 to 16, where it achieved the highest F1-score, indicating optimal model performance. It is noteworthy that having different k values within a relatively large range suggests the robustness of the DFASGCNS model in ovarian cancer prognosis prediction.

### Sensitivity analysis of the attention mechanism

**Fig 3:**
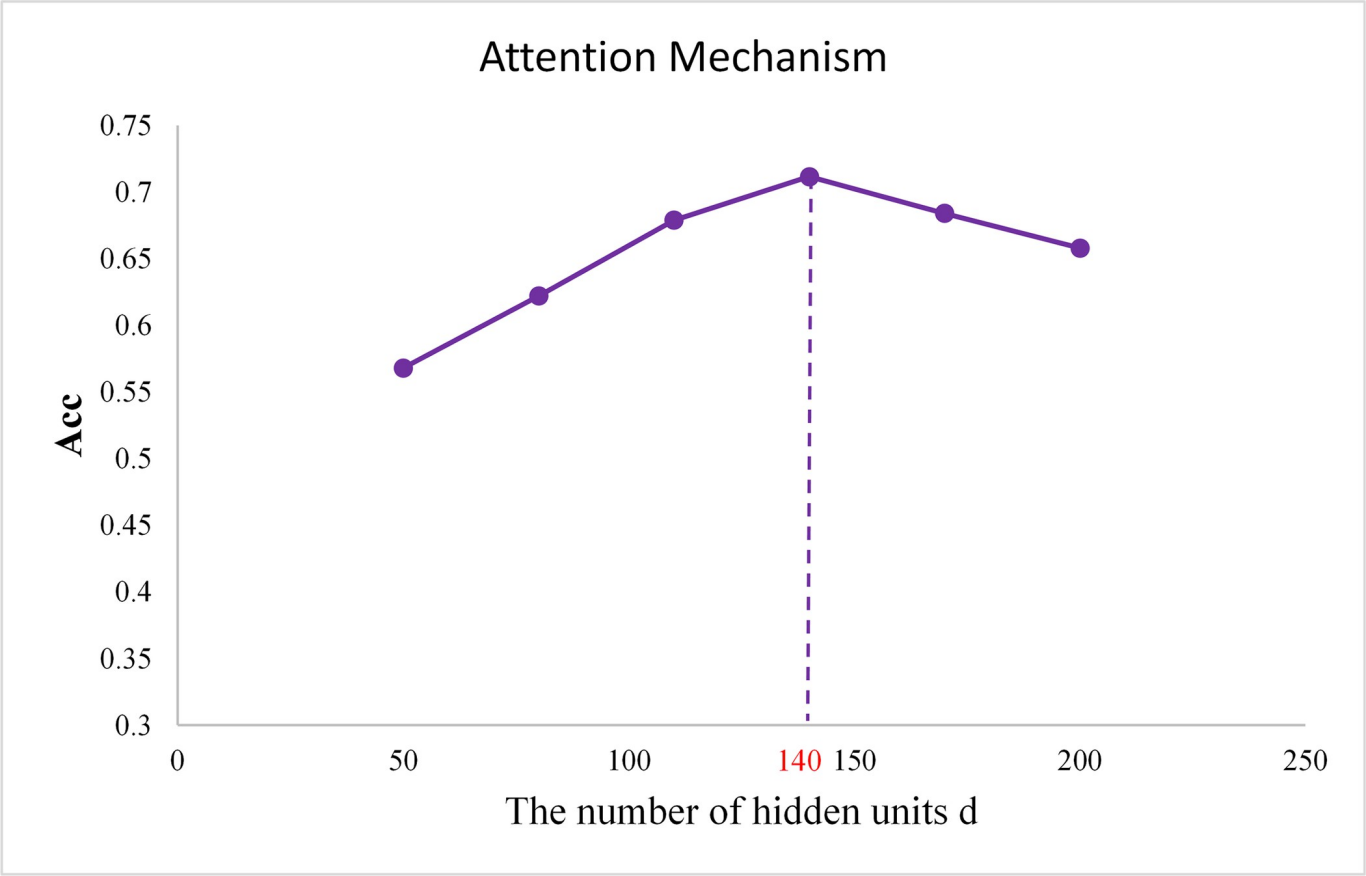
Prognostic prediction of OV by different number of hidden units in attention mechanism.

To evaluate the effectiveness of the attention mechanism in integrating multi-omics data for predicting the prognosis of ovarian cancer patients, we conducted a sensitivity analysis on the number of hidden units d in the attention mechanism. The aim was to reveal how attention mechanisms of different scales affect the model’s performance. In this paper, we used preprocessed mRNA expression, miRNA expression, DNA methylation, and CNV datasets as inputs for DFASGCNS. After RLASSO feature selection, we performed five-fold cross-validation experiments on ovarian cancer prognosis prediction using different numbers of hidden units d in the attention mechanism. The number of hidden units d in the attention mechanism of DFASGCNS was set to 50, 80, 110, 140, 170, and 200, respectively, and validated. The experimental results are shown in Fig 3. In Fig 3, when the number of hidden units in the attention mechanism of DFASGCNS was set to 140, the Acc value reached 71.16%, and the model’s performance was optimal. Thereafter, as the number of hidden units d increased, the Acc showed a downward trend, indicating that an increase in the number of hidden units in the attention mechanism of DFASGCNS may lead to gradient vanishing, reducing the model’s ability to learn important features, thereby affecting the prediction performance.

### Ablation experiment

#### Ablation experiments with different omics data

To investigate the impact of different types of omics data on ovarian cancer prognosis prediction, this study conducted experiments using single-omics data, arbitrary combinations of two types of omics data, arbitrary combinations of three types of omics data, and integration of four omics data sets. The dataset was randomly divided into a 70% training set and a 30% validation set, and the process was repeated 10 times. The results are presented in Tables 4 and 5.

**Table 4:** The prognostic prediction results of ovarian cancer using single omics data and arbitrary integration of two omics data (%).

mRNA	√				√	√	√			
DNA methylation		√			√			√	√	
miRNA			√			√		√		√
CNV				√			√		√	√
ACC	62.26	59.81	56.28	59.45	64.32	62.47	62.89	61.55	63.36	60.92
F1-score	63.92	61.17	59.76	60.24	69.70	66.19	65.32	61.93	64.70	62.57
AUC	60.59	58.70	56.52	58.99	62.19	60.73	60.68	60.28	59.51	58.99

**Table 5:** The prognostic prediction results of ovarian cancer using arbitrary integration of three omics data and integration of four omics data (%).

mRNA	√	√	√		√
DNA methylation	√	√		√	√
miRNA	√		√	√	√
CNV		√	√	√	√
ACC	67.59	68.38	65.71	64.96	**71.16**
F1-score	73.72	75.34	72.98	71.23	**79.25**
AUC	63.13	63.29	62.76	60.60	**64.77**

From Table 4, it is evident that different types of omics data exhibit distinct performance in ovarian cancer prognosis prediction. Specifically, when using a single type of omics data, mRNA expression demonstrates the best performance, followed by DNA methylation, while miRNA expression and CNV perform the poorest. This difference reflects the varying roles of different omics molecular information in ovarian cancer development and prognosis [35,36]. When integrating any two types of omics data, combining mRNA expression and DNA methylation yields the best results, while integrating miRNA expression and CNV data yields the worst results. Furthermore, the prognostic predictions based on mRNA expression combined with other omics data are superior to those of integrating two types of omics data without mRNA expression. This indicates that mRNA expression contributes most prominently to ovarian cancer prognosis prediction, followed by DNA methylation, while the contributions of mRNA expression and CNV are relatively minor, consistent with previous research findings [37].

The results in Table 5 demonstrate that integrating four types of omics data yields the best performance in ovarian cancer prognosis prediction, with ACC, F1-score, and AUC values of 71.16%, 79.25%, and 64.77%, respectively. Among the combinations of any three types of omics data, the combination of mRNA expression, DNA methylation, and CNV follows the integration of four types of omics data, with performance indicators of ACC, F1-score, and AUC decreasing by 2.78%, 3.91%, and 1.48%, respectively. Comparatively, integrating DNA methylation, miRNA expression, and CNV exhibits the largest decrease in each evaluation metric compared to integrating four types of omics data, with ACC, F1-score, and AUC decreases of 6.20%, 8.02%, and 4.17%, respectively. This indicates that the removal of mRNA expression data has the most significant impact on model performance, further validating the importance of mRNA expression data. Combining the analyses from Tabled 4 and 5 reveals that integrating multiple types of omics data significantly outperforms single or partial omics data, underscoring the importance of complementary nature and comprehensive use of multi-omics data to enhance ovarian cancer prognosis prediction. Consequently, the comprehensive consideration of multiple types of omics data would provide stronger support for personalized treatment in clinical practice and the formulation of ovarian cancer management strategies [2].

#### Ablation experiments of model structures

To validate the contributions of each module in the proposed model DFASGCNS to ovarian cancer prognosis prediction, we conducted ablation experiments by altering different parts of the model configuration. We evaluated the importance of each module in the DFASGCNS model using the following five different configurations:

- SGCN: Constructing feature graphs using single omics data that underwent RLASSO feature selection and applying SGCN on each single omics graph.
- NoAttention-SGCN: Predicting using fused features after RLASSO and fusion graph *A*, concatenating the low-dimensional features *Z*^(1)^、*Z*^(2)^、*Z*^(3)^ and *Z*^(4)^ of the four types of omics data without including an attention layer.
- NoAttention-FF: Employing only fused features after RLASSO and fully connected layers for prediction, concatenating the low-dimensional features *Z*^(1)^、*Z*^(2)^、*Z*^(3)^ and *Z*^(4)^ of the four types of omics data without attention layers and SGCN module.
- Attention-FF: Utilizing fused multi-omics features with added attention mechanism and fully connected layers for prediction without the SGCN module.
- Without-Graph: Removing the graph from the entire model structure and replacing it with an identity matrix in SGCN.

This paper adjusted different parts of the ovarian cancer dataset to implement the five variants of DFASGCNS mentioned above. Among them, SGCN is a single-omics method, NoAttention-SGCN uses a feature fusion method without attention mechanism, removing the attention mechanism from DFASGCNS. NoAttention-FF is a feature fusion method without attention mechanism, and a fully connected network is introduced after advanced feature fusion, removing the attention mechanism and SGCN from DFASGCNS. Attention-FF is based on NoAttention-FF, using a feature fusion method with attention mechanism, removing only SGCN from DFASGCNS. Without-Graph is a DFASGCNS without graph structure, replacing the fusion graph A in DFASGCNS with an identity matrix. For fair comparison, the same neural network is used to extract features of omics data in the above five configurations, as shown in Table 6.

**Table 6:** The predictive results using different variant model architectures (%).

Omics data type	Method	ACC	F1-score	AUC
mRNA	SGCN	63.28±1.31	64.16±1.87	60.41±1.34
DNA methylation		59.92±1.62	61.32±1.26	57.98±0.97
miRNA		56.78±1.75	59.11±1.60	57.12±1.54
CNV		59.53±1.84	60.59±1.54	58.81±1.04
Integrate four types of omics data	NoAttention-SGCN	69.95±1.57	78.62±1.68	64.08±1.25
	NoAttention-FF	65.46±1.77	73.94±1.42	62.53±1.84
	Attention-FF	66.83±1.59	75.03±1.39	62.97±1.75
	Without-Graph	65.38±1.24	74.65±1.80	62.17±1.63
	DFASGCNS	**71.16**±**1.52**	**79.25**±**1.32**	**64.77**±**1.26**

### Comparative experiments with existing methods

To evaluate the effectiveness of the DFASGCNS model in predicting the prognosis of ovarian cancer, this paper compared the DFASGCNS model with existing traditional and deep learning-based prognostic prediction methods. Among them, SVM [38], RF [39], and XGBoost [40] utilize machine learning methods to integrate multi-omics data for prognostic prediction of ovarian cancer, while MDNNMD [41], GCGCN [16], MOLI [42], DeepMO [19], MOGONET [17], MOCSC [43], and MDCADON [44] are all deep learning methods. Label predictions were made by a majority vote of KNN in the training data, with K set to 37 to minimize errors. In RF, multiple decision trees are combined through ensemble learning to obtain the final prediction results. XGBoost employs gradient boosting technique for early and late-stage cancer classification. MDNNMD integrates patient prognosis prediction results through score fusion. GCGCN adopts graph fusion method, while MOLI, DeepMO, MOGONET, MOCSC, and MDCADON adopt late fusion methods for multi-omics data. Among them, DeepMO, MOGONET, MOCSC, and MDCADON utilize chi-square test, GCN, DAE, and RLASSO feature selection algorithms for gene screening. To comprehensively evaluate the proposed DFASGCNS model, this study employed repeated hold-out cross-validation following the method proposed by Lee et al. [45] The dataset was randomly divided into 70% training set and 30% testing set. Different methods were trained on the training set to build prediction models, and evaluation metrics were calculated by predicting survival on the test patients. The experiments were repeated 10 times, and the results are shown in Table 7.

**Table 7:** The results of prognosis prediction for ovarian cancer using DFASGCNS compared to existing methods (%).

Method	ACC	F1-score	AUC
SVM	54.28±1.87	53.59±1.66	54.43±2.25
RF	55.16±3.49	54.17±2.36	53.12±2.11
XGBoost	56.04±2.12	54.66±1.60	54.92±0.87
MDNNMD	60.85±1.88	68.42±2.03	57.22±1.89
GCGCN	64.52±1.75	71.19±1.64	61.71±1.53
MOLI	63.08±1.21	70.94±1.80	59.48±1.62
DeepMO	63.98±1.72	70.25±1.69	60.17±1.23
MOGONET	64.25±2.36	73.16±2.20	57.94±1.98
MOCSC	65.48±1.83	73.45±2.31	59.25±2.17
MDCADON	69.47±2.10	77.91±1.82	63.45±2.15
**DFASGCNS**	**71.16**±**1.52**	**79.25**±**1.32**	**64.77**±**1.26**

In Table 7, it can be observed that the proposed model DFASGCNS achieves the highest values in evaluation metrics Acc, F1-score, and AUC, with values of 71.16%, 79.25%, and 64.77% respectively. Compared to other methods, it demonstrates better accuracy in predicting the prognosis of ovarian cancer patients. This is attributed to the consideration of inter-sample correlations in ovarian cancer within the multi-omics feature fusion of the DFASGCNS model. By utilizing SGCN to learn the deep network structure of the dual fusion channel and introducing attention mechanism during feature fusion of different omics data, DFASGCNS adequately addresses the importance of different omics features, thus enhancing the model’s performance in predicting ovarian cancer prognosis. The relatively high F1-score value of the DFASGCNS model is due to its weighted combination of precision and recall, which provides relatively accurate results even in the presence of imbalanced datasets [46]. Overall, the evaluation metrics of deep learning methods are higher than those of machine learning methods (SVM, RF, and XGBoost), indicating that deep learning methods can more fully learn the high-dimensional features of multi-omics data related to ovarian cancer and extract important information. Among the deep learning-based methods for integrating multi-omics data, DeepMO improves upon MOLI by incorporating a feature selection method, leading to improvements in evaluation metrics Acc and AUC, underscoring the importance of feature selection in ovarian cancer prognosis prediction. Both MOGONET and DFASGCNS utilize GCN to learn advanced features of omics data. However, DFASGCNS not only considers features of different omics data but also incorporates the graph structure of relationships between ovarian cancer samples, facilitating a comprehensive exploration of inter-sample correlations and thereby enhancing ovarian cancer prognosis prediction performance. Furthermore, compared to the MDCADON model, the DFASGCNS shows improvements in all evaluation metrics. In summary, compared to existing methods, the DFASGCNS model presented in this study can more effectively learn feature representations of multi-omics data and correlations between samples, thereby improving the accuracy and reliability of predicting ovarian cancer prognosis.

### External validation of GEO datasets

**Fig 4:**
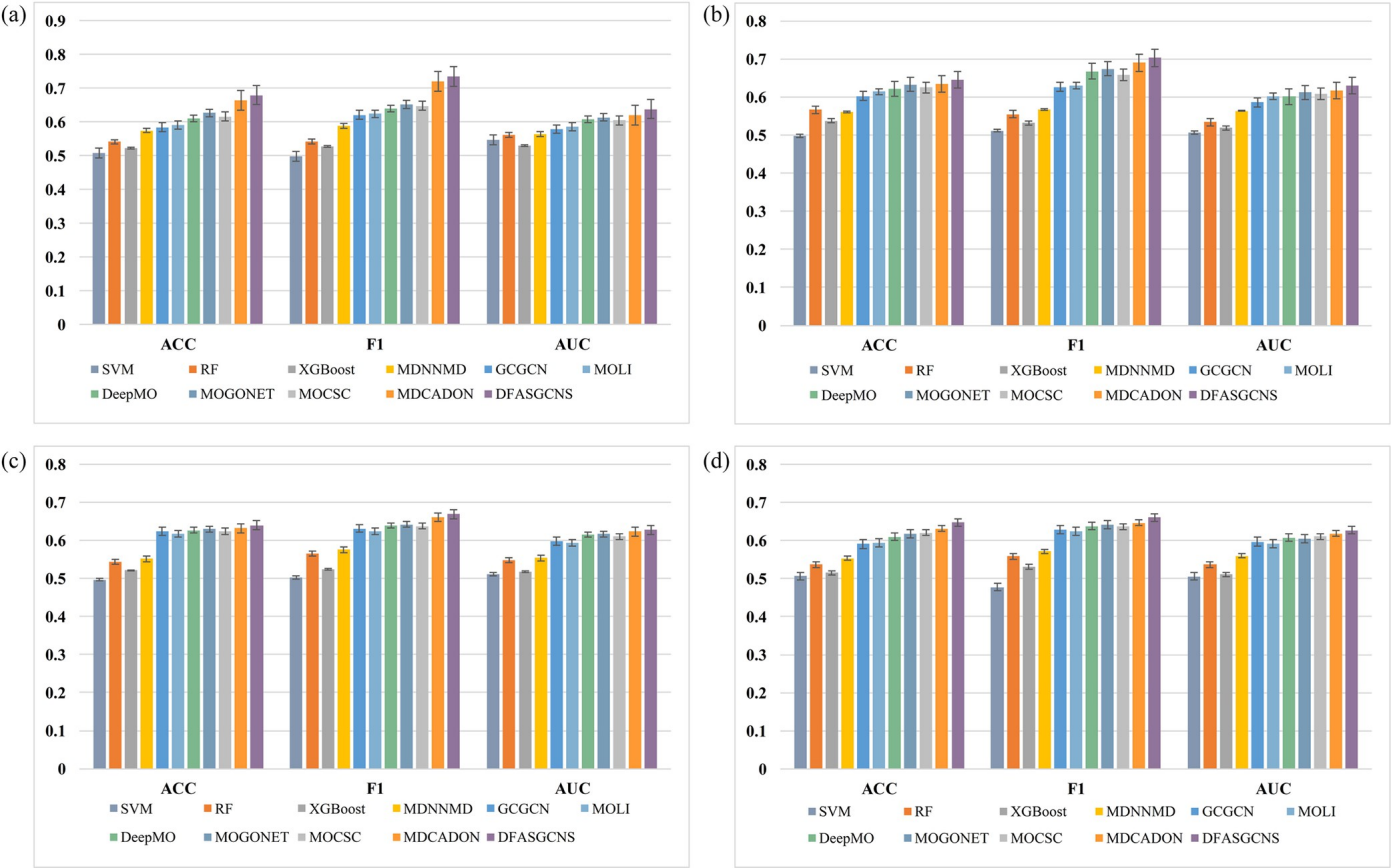
The results of DFASGCNS compared to other existing methods on the GEO datasets. (a) GSE26712 dataset. (b) GSE32062 dataset. (c) GSE17260 dataset. (d) GSE140082 dataset.

To investigate the generalization performance of the DFASGCNS model across different datasets, this study utilized four GEO datasets of ovarian cancer, including GSE26712 [47], GSE32062 [48], GSE17260 [49], and GSE140082 [50]. Detailed information is provided in Table 8. To ensure the objectivity and comparability of the experimental results, the GEO datasets were randomly divided into 70% training set and 30% testing set, and this process was repeated 10 times to ensure the independence and randomness of training and testing data. The DFASGCNS model was compared with existing methods, and the results are illustrated in Fig 4.

**Table 8:** The properties of the GEO datasets.

Datasets	Sample numbers	Data category	Gene annotation platform
GSE26712	185	RNA-seq	GPL96 Affymetrix
GSE32062	260	Gene expression	GPL6480 Agilent
GSE17260	110	Gene expression	GPL6480 Agilent
GSE140082	380	Gene expression	GPL14951 Illumina

From Fig 4, it is evident that across the four GEO datasets, the evaluation metrics of the DFASGCNS model are higher than those of other methods. Specifically, in the GSE26712 dataset, the predictive performance of deep learning methods surpasses that of machine learning methods, the DFASGCNS model achieving good predictive results in terms of evaluation metrics ACC and F1-score. Similarly, when training the DFASGCNS model on the GSE32062, GSE17260, and GSE140082 datasets and evaluating it on the test sets, the DFASGCNS model exhibits superior predictive capabilities compared to other methods. Compared to machine learning methods, the performance of the DFASGCNS model is significantly improved, while the improvement is less pronounced compared to deep learning methods. This phenomenon can be attributed to the fact that these four GEO datasets only contain single types of omics data, while the DFASGCNS model proposed in this study is capable of predicting on multiple omics data. Therefore, its performance improvement on GEO datasets is relatively modest. Nevertheless, the DFASGCNS model still achieves good performance even on single-omics data, comparable to methods like MOLI and DeepMO, indicating its good generalization ability and stable and excellent predictive performance across different datasets. This provides greater reliability and feasibility for clinical practice in ovarian cancer prognosis prediction. Thus, the DFASGCNS model proposed in this study not only performs well on multi-omics data but also demonstrates good performance on single-omics data, further validating its effectiveness in ovarian cancer prognosis prediction.

### Survival analysis

**Fig 5:**
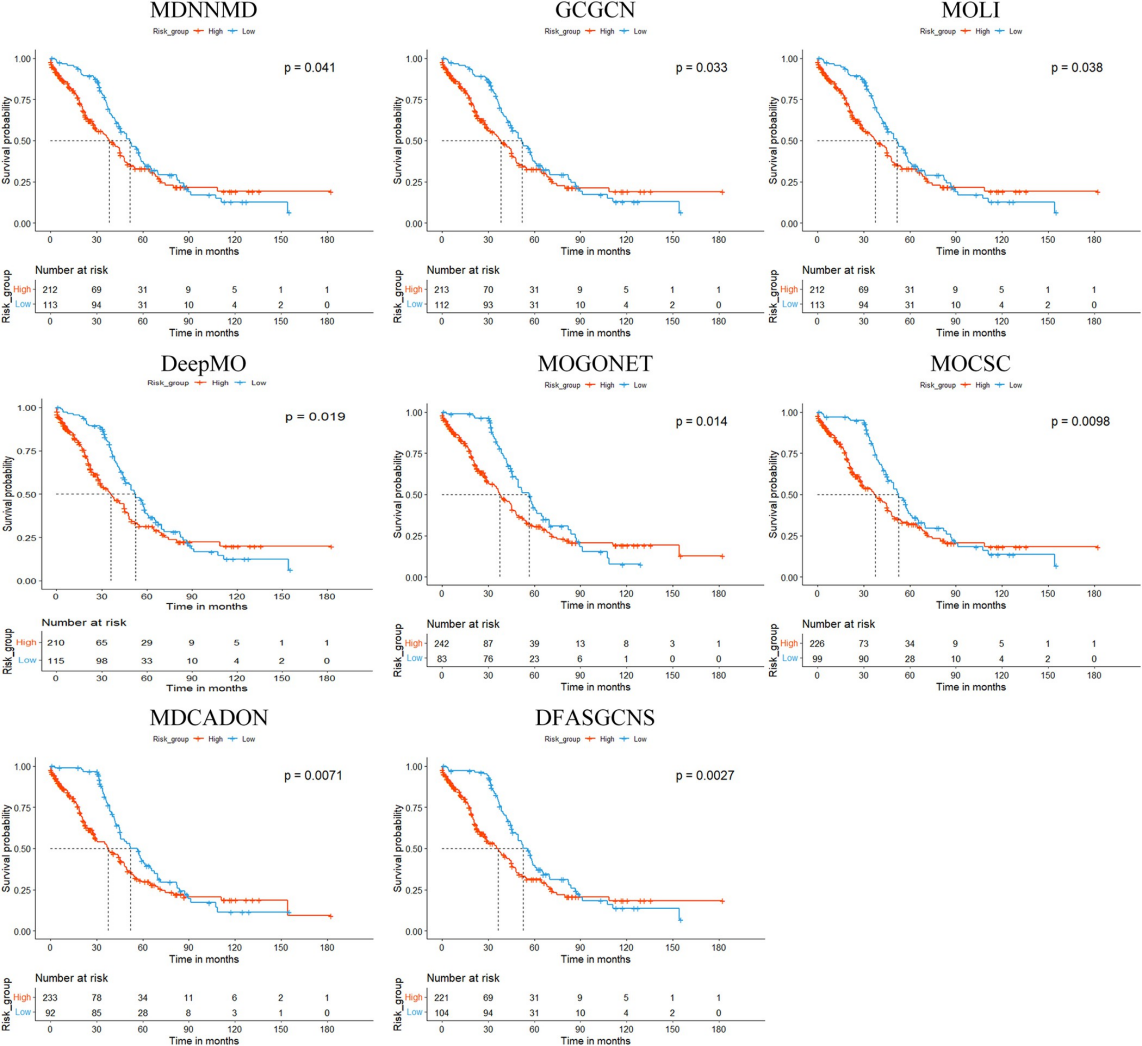
The Kaplan-Meier survival curves of ovarian cancer patients generated by different methods.

To further evaluate the performance of DFASGCNS, logrank tests were conducted to predict whether patients in high-risk and low-risk subgroups exhibit significantly different survival curves. Specifically, using the threshold of the median risk ratio, patients in the ovarian cancer dataset were divided into high-risk and low-risk subgroups based on the predicted risk ratios. Subsequently, a log-rank test was performed to evaluate whether there is a significant difference in the actual survival times between the two sample groups. The p-value serves as a crucial indicator for assessing the statistical significance of differences in survival data between groups. A p-value of less than 0.05 indicates statistical significance, with smaller p-values suggesting more pronounced differences between the two subgroups, thereby reflecting a more effective prediction method. In this study, Kaplan-Meier survival curves were plotted based on the ovarian cancer prognosis predictions from all deep learning methods used in the comparison. The log-rank p-values corresponding to each method were obtained, as shown in Fig 5. The x-axis represents time, and the y-axis represents the survival rate, as depicted in Fig 5.

From Fig 5, it can be observed that all deep learning methods applied to ovarian cancer patient survival prognosis yield p-values less than 0.05, indicating significant effectiveness in prognostic prediction. By comparing with seven existing methods, it was found that the model DFASGCNS proposed in this study generated the most significant p-value (*p* = 0.0027). This result indicates that the DFASGCNS model achieves the best performance in survival prediction, with the survival differences between the high-risk and low-risk subgroups being more pronounced and highly significant. This highlights the superiority of the model in predicting ovarian cancer survival outcomes.This finding further emphasizes the importance and effectiveness of the DFASGCNS model in ovarian cancer survival prediction. By introducing a dual-channel fusion strategy in deep learning methods, which learns feature representations of multiple omics data and shares and complements neighborhood information among samples, and by employing attention mechanism to assign corresponding attention weights to important features of different omics data, thereby optimizing the feature representation of multiple omics data. The DFASGCNS can more accurately capture survival differences among patients and provide more discriminative predictive results. This is significant for clinical practice and treatment decision-making, as it helps doctors better understand the prognosis of patients and thus develop more effective treatment plans.

### Enrichment of selected genes

**Fig 6:**
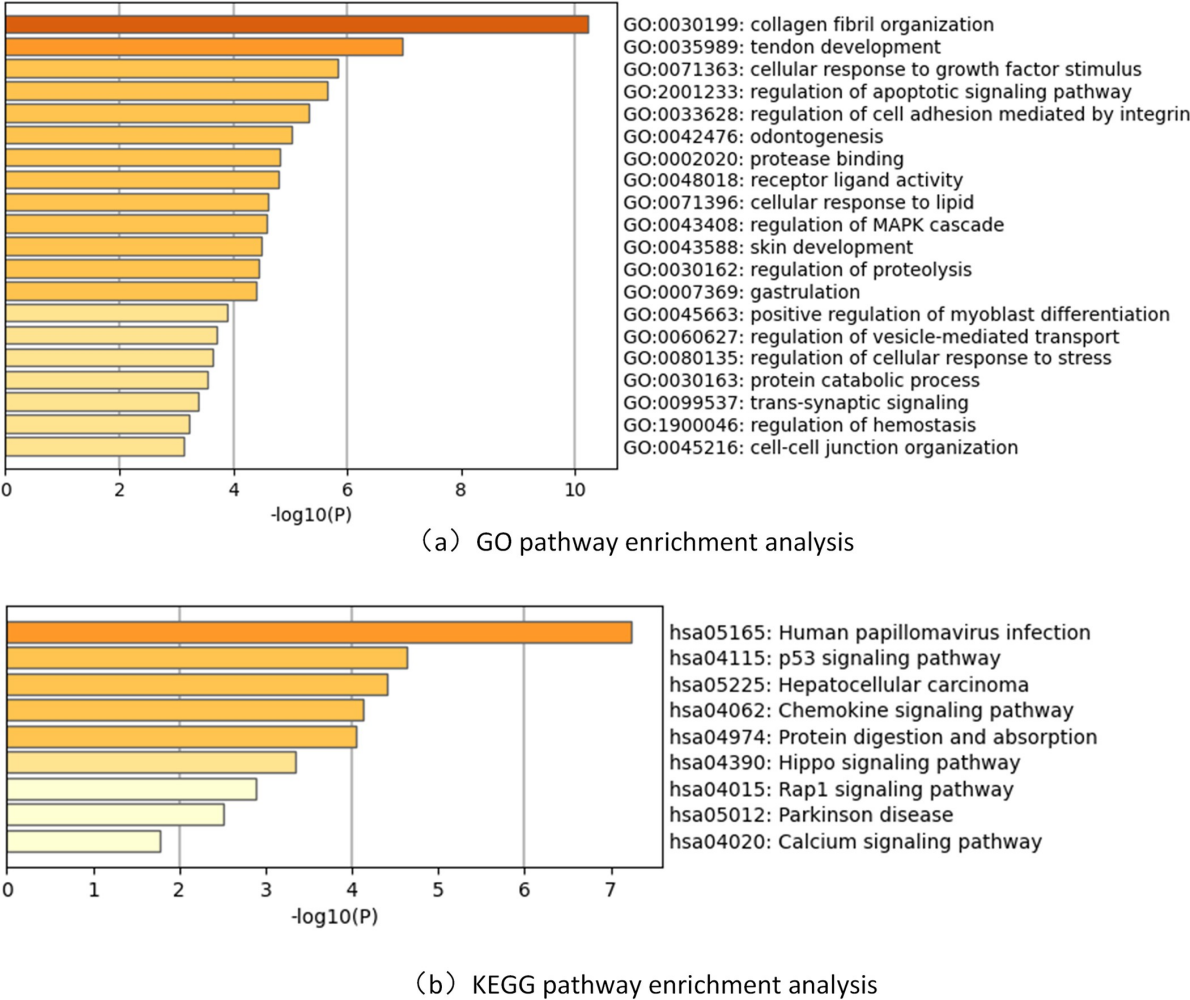
Pathway enrichment analysis of identified genes. The x-axis represents the -log10 p-value for each term, and the y-axis represents the KEGG pathway terms. (a) GO pathway enrichment analysis. (b) KEGG pathway enrichment analysis.

To further investigate the impact of multi-omics data features on the pathogenesis of ovarian cancer, we employed the RLASSO feature selection algorithm to identify omics data features. These features were ranked by their importance using random forests, and the top 20 important gene features were subjected to GO (Gene Ontology) and KEGG (Kyoto Encyclopedia of Genes and Genomes) pathway analysis via the online tool Metascape, as shown in Fig 6. The top 20 important gene features selected in this study include: ADH1B, USP25, VDR, STK17B, TPST2, CXCL9, PTPRC, CXCL14, TP53, CXCL11, KRAS, PBK, NRGN, SCNN1A, POLD2, POLR1C, SAR1A, PTEN, NRAS, ZNF826P.

Among them, ADH1B encodes a protein that is a member of the alcohol dehydrogenase family, and it has been proven to promote mesothelial clearance and ovarian cancer infiltration [51]. CXCL9 has been confirmed to be associated with ovarian cancer prognosis; it is related to the survival rate of patients with high-grade serous ovarian cancer (HGSC) and is an independent marker of good prognosis in HGSC patients [52]. In ovarian cancer patients, abnormal overexpression of CXCL14 in serum and ovarian tissues is associated with poor prognosis, making it a novel auxiliary marker for early diagnosis of ovarian cancer [53]. TP53 mutations are closely associated with high recurrence rates, chemotherapy resistance, and shorter survival in ovarian cancer [54]. CXCL11 is highly expressed in the immunoreactive subtype, and its ligand CXCL11 and receptor CXCR3 characterize this subtype, with the CXCL11-CXCR3 signaling pathway being a therapeutic target for ovarian cancer [55]. KRAS mutations are associated with low-grade serous ovarian cancer, often predicting poor prognosis, particularly in patients with poor chemotherapy response [56]. PTEN is a tumor suppressor gene that negatively regulates the PI3K/AKT signaling pathway. Studies have shown that loss or mutation of PTEN is associated with increased invasiveness, enhanced anti-apoptosis, and poorer prognosis in ovarian cancer [57]. From Fig 6, GO/KEGG enrichment analysis revealed several important pathways associated with ovarian cancer, providing valuable insights into the mechanisms of ovarian cancer occurrence, metastasis, and treatment. Among these, pathways such as vesicle-mediated transport and human papillomavirus (HPV) infection are related to the development and metastasis of ovarian cancer. Extracellular vesicles play a significant role in cell-to-cell communication and have been implicated in tumor formation and metastatic disease [58]. Additionally, the identified pathway related to HPV infection has been shown to be highly associated with ovarian cancer [59]. The Hippo signaling pathway is a highly conserved pathway that regulates organ size and plays a key role in ovarian physiology. Dysregulation of the Hippo pathway contributes to loss of follicular homeostasis and reproductive disorders such as polycystic ovary syndrome (PCOS), premature ovarian insufficiency, and ovarian cancer [60]. Furthermore, we also identified pathways associated with other cancers or diseases, including hepatocellular carcinoma and Parkinson’s disease [37]. This helps to elucidate the connections between ovarian cancer and other diseases, providing important clues for deeper exploration of ovarian cancer pathophysiology.

### Generalizability across different cancer types

To validate the generalization capability of the DFASGCNS model, this study conducted comparative experiments using single-omics and multi-omics data on the Lower Grade Glioma (LGG) and Lung Squamous Cell Carcinoma (LUSC) datasets. In the experiments, four types of omics data (mRNA, DNA methylation, miRNA, and CNV) were used for LGG and LUSC. The DFASGCNS model first employed the RLASSO feature selection method to extract key features, which were then integrated using an attention mechanism. For each type of omics data, the model constructed corresponding graph structures to capture the relationships between samples and underlying network structures. By stacking multiple layers of graph convolutional networks, the model further learned deep correlations and complex relationship networks among multi-omics samples. Finally, a Softmax classifier was used for prognosis prediction of LGG and LUSC. Ten-fold cross-validation was performed, using Acc, F1-score, and AUC as evaluation metrics. The results of Acc, F1-score, and AUC for single-omics and multi-omics data on LGG and LUSC are shown in Table 9. As seen from Table 9, compared to single-omics prognosis prediction, the AUC, Acc, and F1-score for multi-omics data on LGG and LUSC all improved, demonstrating the effectiveness of the DFASGCNS model in integrating multi-omics data.

**Table 9:** Prognostic results of different cancer datasets (%).

Datasets	mRNA	√			√	√		√
	meth		√		√		√	√
	miRNA			√		√	√	√
	Acc	62.12	61.41	60.93	63.36	62.43	62.80	70.44
LGG	F1-score	65.31	62.80	61.35	68.54	65.91	63.52	73.62
	AUC	60.10	59.44	57.14	61.24	60.78	59.91	62.76
LUSC	Acc	63.43	62.52	62.02	66.91	65.04	64.73	71.11
	F1-score	66.21	63.11	62.93	70.12	69.90	68.56	74.80
	AUC	61.22	58.96	58.31	62.25	61.72	60.42	63.03

Additionally, further comparative experiments were conducted between the DFASGCNS model and other methods on the LGG and LUSC datasets, as shown in Table 10. The results indicated that the DFASGCNS model outperformed the other methods in terms of AUC, Acc, and F1-score on both datasets. Among the comparison methods, SVM, RF, and XGBoost are machine learning methods, while the others are deep learning methods. As shown in Table 10, deep learning methods achieved better performance than machine learning methods on the LGG and LUSC datasets, demonstrating the superiority of deep learning for cancer prognosis prediction. Specifically, for LGG prognosis prediction, the DFASGCNS model achieved AUC, Acc, and F1-score values of 70.44%, 73.62%, and 62.76%, respectively. For LUSC prognosis prediction, the AUC, Acc, and F1-score values were 71.11%, 74.80%, and 63.03%, respectively. These results indicate that the DFASGCNS model effectively integrates multi-omics data and achieves favorable cancer prognosis prediction performance, further validating the effectiveness and generalizability of DFASGCNS in predicting the prognosis of different cancer types.

**Table 10:** Comparison of prognostic results of different methods on different cancer datasets (%).

Method	LGG			LUSC		
	Acc	F1-score	AUC	Acc	F1-score	AUC
SVM	60.11	63.54	57.90	62.88	65.52	59.25
RF	59.85	64.75	57.13	63.59	64.16	58.19
XGBoost	62.42	64.11	58.22	63.13	65.95	59.36
MDNNMD	65.12	67.21	59.10	64.92	67.21	60.93
GCGCN	64.93	66.96	59.37	65.75	66.49	61.14
MOLI	68.26	68.30	58.68	65.24	69.23	60.74
DeepMO	68.70	67.52	60.33	68.46	68.91	61.62
MOGONET	69.41	69.11	61.46	67.43	70.32	62.05
MOCSC	68.72	70.43	60.86	69.15	71.14	61.93
MDCADON	69.15	72.82	61.95	70.27	72.93	62.40
DFASGCNS	70.44	73.62	62.76	71.11	74.80	63.03

## Conclusion

This paper proposes an ovarian cancer prognosis prediction model, DFASGCNS, which uses a dual fusion channel and stacked graph convolutional network (SGCN). The introduction of attention mechanism explores the importance of key features from different omics data for ovarian cancer prognosis prediction. To capture the correlation between different omics data and fully consider the inter-sample relationships in ovarian cancer, a dual fusion channel strategy is proposed, enabling comprehensive learning of feature representations of multiple omics data and the sharing and complementing of neighborhood information among samples. The use of SGCN learns the fused features of multiple omics data and the latent network structure between samples, facilitating a comprehensive understanding of the complex relationship network in ovarian cancer multi-omics data. Additionally, external validation of the DFASGCNS model was conducted using four GEO datasets of ovarian cancer. The results demonstrate that learning feature representations of multiple omics data and the relationship graph between samples using a dual fusion channel can effectively learn high-dimensional feature representations of multiple omics data from different perspectives. The utilization of SGCN to capture the latent network structure of multiple omics data significantly improves the accuracy of ovarian cancer prognosis prediction. Furthermore, Kaplan-Meier survival curves show significant differences in survival subgroups predicted by DFASGCNS, contributing to an in-depth understanding of the pathogenesis of ovarian cancer and to the search for new therapeutic strategies.

Although the proposed model achieves promising performance in ovarian cancer prognosis prediction, there are still some areas for improvement. In future work, we plan to incorporate histopathological images of ovarian cancer into the multi-omics data, leveraging image information for comprehensive research on ovarian cancer and providing a new research approach for ovarian cancer prognosis prediction.
